# Hooper’s Physician’s Vade Mecum

**Published:** 1884-09

**Authors:** 


HOOPER'S PHYSICIAN'S VADE MECUM: *A Manual of the Principles and Practice of Physic; with an Outline of General Pathology, Therapeutics and Hygiene. Tenth Edition. Revised by* WILLIAM AUGUSTUS GUY, M. B. CANTAB, F. R. S., *and* JOHN HARLEY, M. D., *Lond.*, F. L. S. *Vol. I., 8 vo., pp., ix., 338. May Number, Wood's Library of Standard Medical Authors. New York:* WILLIAM WOOD & CO.; *Chicago:* W. T. KEENER. 1884.

We heartily agree with the *dictum* of the American publishers, that "One of the remarkable books in medicine is Hooper's 'Physician's Vade Mecum.'" It is a poor book of a very bad class of books. In one volume of three hundred and thirty-eight pages is contained an abstract of our knowledge of General Physiology, Pathology, Therapeutics, and General Diseases! Typographical errors are numerous and the illustrations are wretched. As if to atone for the general poverty of the book, the names of Doctors Guy and Harley appear as revisers on the title page. The gullibility of the American medical practitioner may be prodigious, but it has certain limits. We protest against the *foisting* of such books upon the medical public by publishers, who have won professional confidence by honorable means.

Hooper's "Physician's Vade Mecum" will find no place in a judiciously selected medical library. W. W. J.

